# Open Versus Closed Kinetic Chain: Exercise Effects on Center of Pressure and Y-Balance in Middle-Aged Women with Knee Osteoarthritis—A Randomized Controlled Trial

**DOI:** 10.3390/healthcare13172173

**Published:** 2025-08-30

**Authors:** June Kang, Ja Yeon Lee, Il Bong Park

**Affiliations:** Department of Sports Rehabilitation, Busan University of Foreign Studies, Busan 46234, Republic of Korea; qkaqkekr@naver.com (J.Y.L.); fnjboss@bufs.ac.kr (I.B.P.)

**Keywords:** knee osteoarthritis, closed kinetic chain exercise, center of pressure, Y-balance test, middle-aged women, dynamic balance, postural stability

## Abstract

**Objective**: Head-to-head evidence comparing closed-kinetic-chain (CKC) and open-kinetic-chain (OKC) training on balance in middle-aged women with knee osteoarthritis (KOA) is limited. Purpose: To compare 10-week hip abduction/external rotation-focused CKC versus OKC on static and dynamic balance. **Methods**: Twenty-two women with KOA were randomized to CKC (n = 11) or OKC (n = 11) and trained twice weekly for 10 weeks. The primary outcome was the center of pressure (COP) during single-leg stance (AP/ML range, excursion, velocity, and RMS); the secondary outcome was the Y-Balance Test (YBT) composite score. **Results**: CKC produced significant within-group reductions across all COP variables and significant YBT increases for both affected and unaffected limbs (*p* < 0.05). OKC showed only small changes in select COP indices and no meaningful change in YBT. Post-intervention between-group comparisons consistently favored CKC for AP/ML and total COP excursion/velocity and for the YBT composite score (*p* < 0.05). **Conclusions**: Under weight-bearing conditions, a hip-focused CKC program that promotes multi-joint coordination and co-contraction yields broader and more consistent improvements in postural stability and dynamic balance than OKC in middle-aged women with KOA. These findings support prioritizing CKC when rehabilitation aims include gait and functional stability.

## 1. Introduction

Knee osteoarthritis (KOA) causes chronic pain and diminishes physical function and quality of life [1]. In middle-aged women in particular, overall fitness parameters—including muscular strength, endurance, flexibility, and muscle mass—decline, and this period coincides with marked hormonal fluctuations during the peri- and postmenopausal stages [2,3]. Consequently, decreases in strength and lean mass, along with increases in body fat, raise the prevalence of KOA in the knee joint [4,5]; in this population, reduced lower-extremity strength impairs balance [6], and muscle weakness is closely associated with degenerative joint disease [7].

Previous studies have reported that KOA is characterized by swelling, inflammation, lower-limb muscle weakness, and impaired proprioception, all of which significantly impact balance and gait [8,9]. These deficits reduce dynamic stability and limit the ability to maintain equilibrium [10]. Functional failure of the knee can progress to osteo-arthritic changes through abnormal movement patterns [11], and weakness of the hip abductors—which are important for lower-limb alignment—reduces stability during the stance phase of gait, potentially increasing the risk of medial compartment KOA [12].

Bhatt, Sheth, and Vyas reported that individuals with KOA experience fear and anxiety related to pain and injury, and such concerns are associated with functional performance [13]. KOA occurs predominantly on the medial side of the knee because approximately 60% of body-weight loading is transmitted medially during walking [14]. Excessive or abnormal loading during daily activities such as walking imposes sustained stress on the joint and substantially contributes to the onset and progression of the disease [15,16].

Karimi and Sharifmoradi reported that patients with knee osteoarthritis (KOA) exhibit reduced static and dynamic stability, which increases the risk of falls [17]. In particular, center-of-pressure (COP) variability rises when transitioning from double- to single-limb support, negatively affecting the performance of daily movements [18]. Furthermore, individuals with severe grades of KOA have been shown to demonstrate greater gait asymmetry than those with mild KOA; increased hip load occurs more on the contralateral non-affected limb than on the affected limb, requiring greater strength from the gluteus maximus, gluteus medius, and rectus femoris, which may in turn exacerbate KOA progression in the contralateral knee [19]. Therefore, to prevent pain, falls, and gait asymmetry in middle-aged women with KOA, careful management and interventions targeting both static and dynamic balance are essential.

Quadriceps strengthening protects the knee articular cartilage and helps attenuate joint loads [20,21]. However, activity limitations are frequently observed in the early stage of knee osteoarthritis [22]. Therefore, combining hip abductor strengthening with lower-limb exercises has been reported to be effective for improving knee symptoms and physical function [23]. 

Closed kinetic chain (CKC) exercise is defined as a movement in which the distal segment of the lower extremity is fixed under weight-bearing conditions while the proximal segment moves [24,25]. CKC exercises are widely used in rehabilitation programs and have been reported to generate the force and power necessary for daily activities such as walking, running, and stair climbing [26,27]. Chen et al. reported that greater activation of the hip abductors and external rotators influences patellar alignment, and that compared with open-kinetic-chain (OKC) exercise, CKC exercise induces simultaneous co-contraction of the vastus lateralis, vastus medialis, and gluteus medius, thereby enhancing hip joint stability [28]. Similarly, Boren et al. reported that CKC movements such as the side plank and single-leg squat significantly increase gluteus medius activity [29]. Thomas and colleagues, as well as Scoppa et al., also recommended CKC exercise programs that include hip abductor strengthening for patients with KOA [30,31]. Notably, weakness of hip abduction strength has been reported to correlate with decreased performance during the Y-Balance Test (YBT) [32], which quantitatively assesses dynamic postural control by integrating proprioception, strength, and flexibility, and is widely recognized as a reliable and valid indicator of dynamic balance ability [33].

Although qualitative consensus and some biomechanical evidence support the functional advantages of closed-kinetic-chain (CKC) exercise, randomized controlled evidence that directly compares CKC with open-kinetic-chain (OKC) exercise in middle-aged women while simultaneously assessing center-of-pressure (COP)-based static balance (the primary outcome) and Y-Balance Test (YBT)-based dynamic balance (the secondary outcome) remains limited. Therefore, this study was designed to address the following research question: “In middle-aged women with knee osteoarthritis (KOA), does a 10-week CKC program improve COP stability and YBT performance more than an OKC program?”

We hypothesized that (1) both groups would demonstrate within-group improvements in balance measures over time, and (2) the magnitude of improvement in all COP variables and YBT performance would be greater in the CKC group than in the OKC group. By testing these hypotheses, we aimed to provide randomized comparative evidence on whether a weight-bearing CKC program emphasizing multi-joint coordination and co-contraction, with a focus on hip abduction and external rotation, can produce comprehensive gains in static and dynamic balance in middle-aged women with KOA.

## 2. Materials and Methods

### 2.1. Participants

Participants in this study were recruited through public notices posted at the Lifelong Education Center of B University and a local community center in B Metropolitan City. Eligible participants were middle-aged women who had been clinically diagnosed with knee osteoarthritis (KOA) at a medical institution and had received a physician’s recommendation for exercise therapy.

The primary outcome of this study was the center of pressure (COP), measured using a force plate (AMTI OR6; AMTI, Watertown, MA, USA). The secondary outcome was dynamic balance, assessed with the Y-Balance Test (Functional Movement Systems Inc., Chatham, VA, USA). Based on Scoppa et al. (2013) [30], who reported an effect size of d = 0.7 for COP (mean difference = 0.31 cm·s^−1^, SD = 0.31 cm·s^−1^), the anticipated effect size was set at 0.7. An a priori power analysis with α = 0.05 and statistical power (1 − β) = 0.80 indicated that a minimum of 26 participants was required. Allowing for an expected attrition rate of 10–20%, the final target sample size was determined accordingly.

Participants were randomly allocated by sealed-lot drawing into a closed-kinetic-chain (CKC) exercise group (n = 13) or an open-kinetic-chain (OKC) exercise group (n = 13). During the intervention, 4 participants withdrew due to personal reasons, resulting in a final sample of 22 participants (CKC group: n = 11; OKC group: n = 11). This study was approved by the Institutional Review Board (IRB approval number: P01−202412−01−003).

The inclusion criteria were as follows:

(1) Women aged 50 to <65 years who were clinically diagnosed with unilateral knee osteoarthritis (KOA) and were prescribed exercise therapy by a physician.
(2) Kellgren–Lawrence grade 1–2 out of a maximum of 4, assessed within the past 6 months [34].
(3) Absence of other musculoskeletal diseases or relevant medical history.

The exclusion criteria were as follows:

(1) History of knee surgery within the past year (due to risk of acute inflammation or severe pain).
(2) History of serious cardiovascular disease (e.g., heart failure, myocardial infarction) or neurological, or psychiatric disorders.
(3) Current severe pain or inflammation that would hinder walking or safe participation in exercise.

Homogeneity testing revealed no significant differences between the groups in terms of age (t = 1.051), height (t = 0.263), weight (t = 0.861), and BMI (t = 0.048), confirming that the baseline physical characteristics of the two groups were statistically comparable. The participants’ general characteristics are presented in Table 1.

### 2.2. Experimental Procedures and Measurements

#### 2.2.1. Static Balance

A pretest–post-test design was employed. Static balance was assessed using a force plate (AMTI OR6, AMTI, Watertown, MA, USA) to record center of pressure (COP) displacement. Participants first stood on the affected limb (knee with osteoarthritis) while the contralateral unaffected limb was flexed to approximately 90° at the hip and knee, maintaining a single-leg stance for 40 s. Three successful trials were collected for each limb with adequate rest between trials. After completing the affected-limb trials, participants rested for 3 min and then repeated the procedure on the unaffected limb. COP data were acquired bilaterally to evaluate recovery in the affected limb and potential compensatory patterns in the unaffected limb.

**Figure 1:**
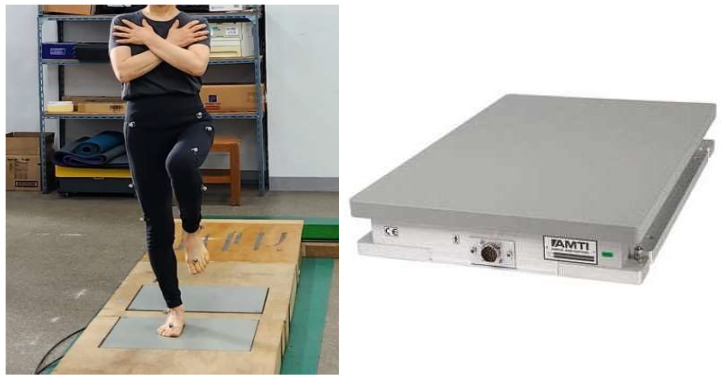
Force plate.

During data collection, COP was sampled for 40 s per trial while participants maintained a single-leg stance on the affected and unaffected limbs. To minimize transient artifacts from initial instability, the first and last 5 s of each trial were excluded from analysis [30]. Time-series analyses were then applied to compute COP variables in the anterior–posterior (AP) and medial–lateral (ML) directions, including range, distance, velocity, and root mean square (RMS). Kinematic data were collected with a Vicon Plug-in Gait motion-capture system (Vicon Motion Systems Ltd., Oxford, UK) at 100 Hz and synchronized with the force-plate signals (Figure 1).

#### 2.2.2. Dynamic Balance

**Figure 2:**
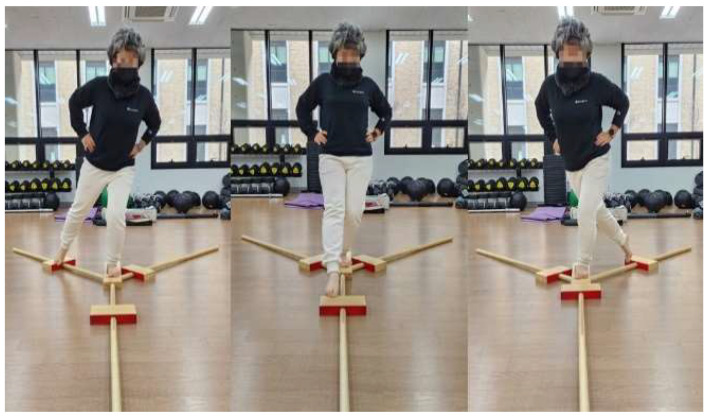
Y-Balance test.

Dynamic balance was assessed pre- and post-intervention using the Y-Balance Test (YBT) kit (Functional Movement Systems Inc., Chatham, VA, USA; Figure 2). Before testing, each participant lay supine in an anatomical neutral position, and the lower limb was aligned so that the hip and knee were positioned along the mid-sagittal axis. Limb length was then measured from the anterior superior iliac spine (ASIS) to the medial malleolus [35].

For testing, participants stood barefoot on the test limb with their hands on the iliac crests and, following standardized instructions, performed reaches with the non-standing limb in three directions: anterior, posteromedial, and posterolateral. A trial was considered invalid if the reaching foot touched on the floor (other than to advance the indicator) or if the participant failed to return to the starting position without a loss of balance; invalid trials were repeated after 30 s of rest [33,36]. Three valid trials were performed in each direction, and the mean reach distance for each direction was recorded. The sequence was as follows: support on the affected limb with the unaffected limb reaching; after a 3 min rest, support on the unaffected limb with the affected limb reaching.

A composite YBT score was then calculated as follows: the sum of the mean reach distances in the anterior, posteromedial, and posterolateral directions was divided by three times the participant’s limb length, and the resulting ratio was multiplied by 100 to express a percentage. This composite value was used as the summary index of dynamic balance performance [37].

### 2.3. Exercise Intervention

#### 2.3.1. Closed-Kinetic-Chain (CKC) Exercise

**Figure 3:**
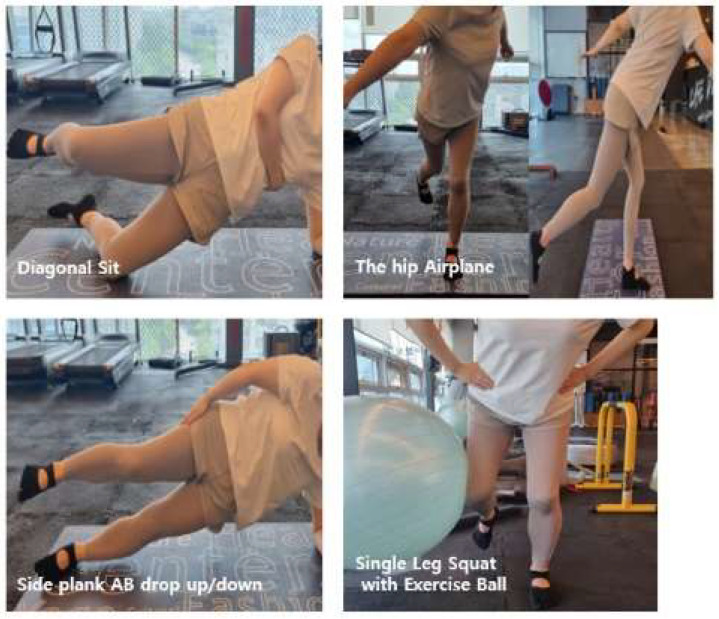
Lower-extremity closed-kinetic-chain exercise.

The CKC intervention was delivered under the joint supervision of an exercise specialist and a licensed physical therapist for 10 weeks, twice weekly (Mondays and Wednesdays), at a standardized time for all participants. Exercise intensity was prescribed using the Borg Rating of Perceived Exertion (RPE) scale, targeting moderate effort 11–13 and adjusted according to individual symptom responses. Each session lasted 60 min and consisted of a 10 min warm-up, 40 min of CKC exercises, and a 10 min cool-down. Following established protocols from prior studies, the program emphasized hip abduction and external rotation, with each exercise performed for 3 sets of 10 repetitions [24,29,38,39,40,41]. The full exercise sequence is illustrated in Figure 3 and detailed in Table 2.

Low Diagonal/Oblique Sit: In a side-lying position, the participant supports their body weight on the bottom elbow and aligns the shoulder–pelvis in a straight line. After performing a small bridge by lifting the bottom pelvis ~2–3 cm, the pelvis is gently rotated toward the support side. Thoracic/lumbar hyperextension is avoided. The difficulty can be modified with a light lift of the bottom foot or an isometric hold (2–3 s up/down; 3–5 s pause at the peak). The participant performs 3 sets × 10 repetitions per side.

Side Plank Hip Abduction Drop/Up: From an elbow side plank, the participant maintains vertical alignment of the ankle–hip–shoulder with the top hand on the pelvis. They abduct the top leg 20–30°, then slowly lower and raise it (2–3 s each), while preventing bottom-side pelvic sag and trunk rotation. They maintain isometric extension/abduction of the bottom hip throughout. They perform 3 sets × 10 repetitions per side.

Single-Leg Squat with Isometric Hip Abduction (Gym ball): Stand on the stance leg with the foot positioned slightly narrower than shoulder width. Lift the contralateral leg and flex the hip and knee to approximately 45°; place a small gym ball between the lateral thigh (near the lateral femoral epicondyle) and the wall; and press the ball laterally to maintain continuous isometric hip abduction. Keeping a neutral spine, perform a single-leg squat through about 30–45° (3 s descent, 1 s hold, and 2 s ascent). Use fingertip support on a wall or rail as needed, tapering assistance as proficiency improves. Perform 3 sets of 10 repetitions per side.

Hip Airplane: From single-leg balance, hinge at the hip to form a “T” alignment, then, while maintaining the hinge, slowly rotate the pelvis–trunk toward and away from the stance side (2–3 s per rotation; 0.5–1 s hold). Keep a straight line from the non-stance leg through the trunk and avoid pelvic drop. Allow fingertip support as needed and withdraw assistance progressively. Perform 3 sets × 10 repetitions per side.

#### 2.3.2. Open-Kinetic-Chain (OKC) Exercise

**Figure 4:**
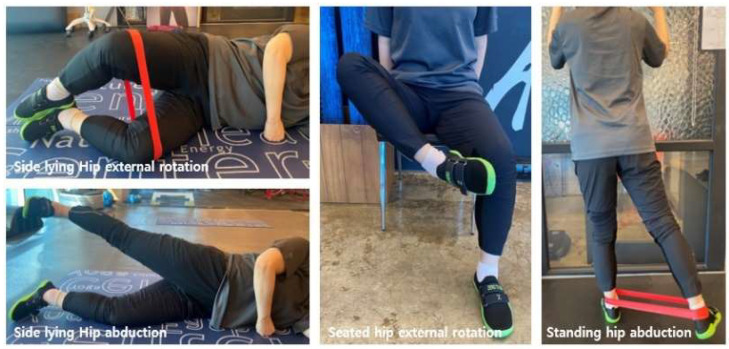
Lower-extremity open-kinetic-chain exercise.

The OKC program was delivered under the joint supervision of an exercise specialist and a licensed physical therapist for 10 weeks, twice weekly (Tuesdays and Thursdays), at a standardized time for all participants. Exercise intensity was monitored using the Borg Rating of Perceived Exertion (RPE), targeting a moderate level 11–13 and adjusted according to individual symptom responses. Each session lasted 60 min and consisted of a 10 min warm-up, 40 min of OKC exercises, and a 10 min cool-down. Following methodologies from prior studies emphasizing hip abduction and external rotation, all exercises were performed for 4 sets of 10 repetitions [29,42]. The full protocol is illustrated in Figure 4 and detailed in Table 3.

Side-lying Hip Abduction: In a side-lying position, maintain a level pelvis and neutral lumbar spine. Raise the top leg in pure abduction to ~20–30°, then lower it along the same path (2–3 s up—1–2 s hold—2–3 s down). Avoid hip hiking, trunk side-bending, and external-rotation compensation.

Side-lying Hip External Rotation (Clamshell): From a side-lying position, with the hips and knees flexed 45–60° and the heels together, externally rotate the upper hip, then return to the starting position at the same tempo. Avoid compensatory movements such as pelvic or trunk rotation.

Seated Hip External Rotation: Sit with the hips and knees at 90°, maintaining a neutral trunk and pelvis. Externally rotate the training-side shank, then return to the original position (at the same tempo). Do not twist only the ankle/foot; avoid knee varus/valgus and trunk rotation.

Standing Hip Abduction: Standing with light support from a wall/rail as needed, maintain neutral alignment and abduct the training limb ~20–30°, then lower it (same tempo). Avoid trunk side-bending, pelvic hiking, and hip flexion/external-rotation compensation.

### 2.4. Data Processing and Statistical Analysis

All statistical analyses were performed using SPSS version 25.0 (IBM Corp., Armonk, NY, USA). Descriptive statistics were presented as means and standard deviations. The Shapiro–Wilk test was conducted to assess normality. In addition, 95% confidence intervals (CIs) were calculated for the main outcome variables to provide estimates of precision alongside *p*-values and effect sizes. To evaluate the intervention effects, a two-way mixed-design ANOVA was performed with time (pre and post) as the within-subject factor and group (experimental, control) as the between-subject factor.

When significant interactions or time-related main effects were observed, post hoc analyses were conducted using paired *t*-tests for normally distributed data and Wilcoxon signed-rank tests for non-normally distributed data. When significant group main effects were found, independent *t*-tests were used for normally distributed data and Mann–Whitney U tests were used for non-normally distributed data. The significance level for post hoc analyses was adjusted using Bonferroni correction at α = 0.05.

## 3. Results

### 3.1. COP Measurements

#### 3.1.1. Comparison of Unaffected Limb COP Parameters Between the CKC and OKC Groups (Table 4)

Following the 10-week intervention, the CKC group showed significant reductions across all COP parameters of the unaffected limb, including the AP range, AP RMS, ML range, ML distance, ML velocity, and Total distance/velocity (all *p* < 0.01, 95% CIs excluding zero), indicating improved balance stability. In contrast, the OKC group exhibited significant post-test increases in AP distance, AP velocity, AP RMS, and ML velocity (all *p* < 0.05), reflecting poorer balance control. Between-group comparisons at post-test further confirmed that the CKC group consistently demonstrated lower COP variability than the OKC group, with significant differences observed in AP distance, AP velocity, ML distance, ML velocity, Total distance, and Total velocity (all *p* < 0.05).

**Table 4:** Comparison of COP parameters in the unaffected limb between CKC and OKC groups.

Variable	Group	Pre	Post	Within-Group (95% CI)	Between Groups (95% CI)	Group (*p*)	Time (*p*)	Interaction (*p*)	η^2^
AP range	CKC	6.01 ± 1.72	0.69 ± 0.07 ***†	+5.33 (3.97, 6.68)	−1.43 (−2.27, −0.59)	0.537	0.000	0.001	0.537
	OKC	4.02 ± 1.50	2.11 ± 1.10 **	+1.91 (0.68, 3.14)					
AP distance	CKC	78.10 ± 20.20	42.64 ± 7.06 **†	+35.47 (18.33, 52.61)	−68.58 (−114.29, −22.86)	0.023	0.569	0.002	0.464
	OKC	62.60 ± 18.11	111.21 ± 59.34 *	−48.61 (−97.81, 0.59)					
AP velocity	CKC	2.60 ± 0.67	1.42 ± 0.24 **†	+1.18 (0.61, 1.75)	−2.29 (−3.81, −0.76)	0.023	0.569	0.002	0.464
	OKC	2.09 ± 0.60	3.71 ± 1.98 *	−1.62 (−3.26, 0.02)					
AP RMS	CKC	0.87 ± 0.19	0.09 ± 0.01 **†	0.78 (0.64, 0.93)	−0.25 (−0.38, −0.11)	0.473	0.000	0.000	0.594
	OKC	0.71 ± 0.17	0.34 ± 0.18 **	+0.38 (0.25, 0.50)					
ML range	CKC	3.99 ± 0.72	0.77 ± 0.09 **†	+3.22 (2.65, 3.78)	−1.74 (−2.64, −0.85)	0.077	0.000	0.000	0.661
	OKC	3.41 ± 0.76	2.52 ± 1.16 *	+0.89 (0.12, 1.67)					
ML distance	CKC	85.56 ± 14.04	46.97 ± 7.54 **†	+38.59 (27.77, 49.40)	−115.38 (−185.78, −44.97)	0.002	0.247	0.002	0.455
	OKC	86.01 ± 18.69	162.35 ± 91.50 *	−76.34 (−147.97, −4.70)					
ML velocity	CKC	2.85 ± 0.47	1.57 ± 0.25 **†	+1.29 (0.93, 1.65)	−3.85 (−6.19, −1.50)	0.002	0.247	0.002	0.455
	OKC	2.87 ± 0.62	5.41 ± 3.05 *	−2.54 (−4.93, −0.16)					
ML RMS	CKC	0.98 ± 0.16	0.08 ± 0.00 ***†	+0.90 (0.78, 1.03)	−0.18 (−0.32, −0.05)	0.776	0.000	0.001	0.527
	OKC	0.76 ± 0.26	0.26 ± 0.18 ***	+0.50 (0.32, 0.68)					
Total distance	CKC	128.29 ± 27.08	71.46 ± 11.69 **†	+56.83 (34.34, 79.32)	−148.28 (−241.80, −54.76)	0.005	0.299	0.002	0.462
	OKC	116.62 ± 28.42	219.74 ± 121.49 *	−103.12 (−199.96, −6.29)					
Total velocity	CKC	4.28 ± 0.90	2.38 ± 0.39 **†	+1.89 (1.14, 2.64)	−4.94 (−8.06, −1.83)	0.005	0.299	0.002	0.462
	OKC	3.89 ± 0.95	7.32 ± 4.05 *	−3.44 (−6.67, −0.21)					

* *p* < 0.05, ** *p* < 0.01, *** *p* < 0.001; within-group difference, † *p* < 0.05; between-group difference at post-test; CKC—closed-kinetic-chain exercise group; OKC—open-kinetic-chain exercise group; COP—center of pressure; AP—anterior–posterior; ML—medial–lateral.

#### 3.1.2. Comparison of Affected Limb COP Parameters Between the CKC and OKC Groups (Table 5)

Following the 10-week intervention, the CKC group showed significant reductions across all COP parameters of the affected limb, including the AP range, AP distance, AP velocity, AP RMS, ML range, ML distance, ML velocity, ML RMS, Total distance, and Total velocity (all *p* < 0.01, 95% CIs excluding zero). In contrast, the OKC group exhibited significant post-test increases in AP distance, AP velocity, AP RMS, ML distance, ML velocity, Total distance, and Total velocity (all *p* < 0.05).

**Table 5:** Comparison of COP parameters in the affected limb between the CKC and OKC groups.

Variable	Group	Pre	Post	Within-Group (95% CI)	Between Groups (95% CI)	Group (*p*)	Time (*p*)	Interaction (*p*)	η^2^
AP range	CKC	5.41 ± 2.52	0.70 ± 0.08 **†	+4.71 (2.75, 6.66)	−1.42 (−2.17, −0.67)	0.650	0.000	0.028	0.266
	OKC	4.50 ± 1.57	2.12 ± 0.98 **	+2.38 (1.32, 3.45)					
AP distance	CKC	74.08 ± 18.91	43.33 ± 6.68 **†	+30.75 (14.24, 47.26)	−71.81 (−122.85, −20.76)	0.017	0.490	0.005	0.393
	OKC	67.07 ± 24.21	115.14 ± 66.30 *	−48.07 (−102.12, 5.98)					
AP velocity	CKC	2.47 ± 0.63	1.44 ± 0.22 **†	+1.03 (0.47, 1.58)	−2.39 (−4.10, −0.69)	0.017	0.490	0.005	0.393
	OKC	2.24 ± 0.81	3.84 ± 2.21 **	−1.60 (−3.40, 0.20)					
AP RMS	CKC	0.91 ± 0.37	0.09 ± 0.01 **†	+0.82 (0.53, 1.11)	−0.25 (−0.38, −0.12)	0.856	0.000	0.003	0.433
	OKC	0.69 ± 0.15	0.34 ± 0.17 **	+0.35 (0.24, 0.47)					
ML range	CKC	4.23 ± 1.43	0.75 ± 0.05 **†	+3.48 (2.38, 4.58)	−1.83 (−2.82, −0.83)	0.238	0.000	0.000	0.552
	OKC	3.31 ± 0.64	2.58 ± 1.30	+0.73 (−0.18, 1.64)					
ML distance	CKC	86.04 ± 8.90	47.16 ± 7.29 **†	+38.88 (30.35, 47.42)	−119.03 (−195.13, −42.92)	0.003	0.219	0.003	0.440
	OKC	83.29 ± 19.58	166.18 ± 98.92 *	−82.89 (−161.70, −4.09)					
ML velocity	CKC	2.87 ± 0.30	1.57 ± 0.24 **†	+1.30 (1.01, 1.58)	−3.97 (−6.50, −1.43)	0.003	0.219	0.003	0.440
	OKC	2.78 ± 0.65	5.54 ± 3.30 *	−2.76 (−5.39, −0.14)					
ML RMS	CKC	0.90 ± 0.20	0.08 ± 0.00 **†	+0.82 (0.67, 0.97)	−0.17 (−0.27, −0.87)	0.417	0.000	0.091	0.168
	OKC	0.87 ± 0.34	0.25 ± 0.13 **	+0.62 (0.40, 0.83)					
Total distance	CKC	124.96 ± 21.48	72.11 ± 11.17 **†	+52.85 (33.20, 72.49)	−154.14 (−256.58, −51.70)	0.005	0.255	0.003	0.424
	OKC	118.00 ± 32.52	226.25 ± 133.12 *	−108.26 (−214.66, −1.86)					
Total velocity	CKC	4.17 ± 0.72	2.40 ± 0.37 **†	+1.76 (1.11, 2.42)	−5.14 (−8.55, −1.72)	0.005	0.255	0.003	0.424
	OKC	3.93 ± 1.08	7.54 ± 4.44 *	−3.61 (−7.16, −0.06)					

* *p* < 0.05, ** *p* < 0.01; within-group difference, † *p* < 0.05; between-group difference at post-test; CKC—closed-kinetic-chain exercise Group; OKC—open-kinetic-chain exercise Group; COP—center of pressure; AP—anterior–posterior; ML—medial–lateral.

Between-group comparisons at post-test further confirmed that the CKC group consistently demonstrated lower variability than the OKC group. Significant group differences were found in AP distance, AP velocity, ML distance, ML velocity, Total distance, and Total velocity (all *p* < 0.05).

### 3.2. Dynamic Balance

#### Single-Leg Balance and Functional Movement (Table 6)

After the 10-week intervention, the CKC group showed significant improvements in Y-Balance Test (YBT) scores for both the affected and unaffected limbs. The affected limb improved from 87.07 ± 8.46 to 100.3 ± 6.23 (95% CI: −17.72 to −7.53, *p* < 0.01), while the unaffected limb improved from 88.60 ± 7.52 to 102.2 ± 3.54 (95% CI: −17.32 to −7.85, *p* < 0.01).

**Table 6:** Comparison of Y-balance test between CKC and OKC groups.

Variable	Group	Pre	Post	Within-Group (95% CI)	Between Groups (95% CI)	Group (*p*)	Time (*p*)	Interaction (*p*)	η^2^
Affected Limb	CKC	87.07 ± 8.46	100.3 ± 6.23 ***†	−12.62 (−17.72, −7.53)	+16.07 (5.84, 26.31)	0.073	0.005	0.000	0.587
	OKC	87.67 ± 10.18	87.29 ± 10.82	2.31 (−2.80, 7.42)					
Unaffected Limb	CKC	88.60 ± 7.52	102.2 ± 3.54 ***†	−12.58 (−17.32, −7.85)	+12.36 (3.65, 21.08)	0.162	0.001	0.000	0.529
	OKC	87.46 ± 11.75	85.15 ± 13.69	+0.38 (−4.28, 5.03)					

*** *p* < 0.001; within-group difference, † *p* < 0.05; between-group difference at post-test; CKC—closed-kinetic-chain exercise group; OKC—open-kinetic-chain exercise group; COP—center of pressure; AP—anterior–posterior; ML—medial–lateral.

In contrast, the OKC group showed no significant changes: the affected limb slightly decreased (87.46 ± 11.75 to 85.15 ± 13.69, 95% CI including zero), and the unaffected limb remained unchanged (87.67 ± 10.18 to 87.29 ± 10.82).

Between-group comparisons at post-test demonstrated significant differences, with the CKC group achieving higher YBT scores for both the affected limb (difference = +16.07, 95% CI: 5.84–26.31, *p* < 0.01) and the unaffected limb (difference = +12.36, 95% CI: 3.65–21.08, *p* < 0.01). Significant group × time interactions were observed in both limbs (*p* < 0.001), confirming that CKC training consistently improved dynamic balance more effectively than OKC training.

## 4. Discussion

This study compared the effects of a 10-week closed-kinetic-chain (CKC) versus open-kinetic-chain (OKC) program—both emphasizing hip abduction and external rotation—on static balance (center of pressure, COP) and dynamic balance (Y-Balance Test, YBT) in middle-aged women with knee osteoarthritis (KOA), using objective balance indices. Following the intervention, the CKC group showed significant improvements in both the primary outcome (COP) and the secondary outcome (YBT), and post-test between-group comparisons confirmed superior balance performance relative to the OKC group.

In middle-aged women, menopausal hormonal changes reduce muscle mass and bone mineral density and alter body composition, thereby increasing KOA risk; these physiological changes coincide with declines in balance and gait capacity [4,5,7]. Accumulating evidence further indicates that impairments in static and dynamic stability in KOA adversely affect daily function, fall risk, and quality of life [17,18]. Although exercise interventions effectively alleviate pain and disability in KOA, prior studies have often prioritized pain and self-reported function, with relatively few concurrently employing objective balance indices such as COP and YBT for comparative evaluation [1,9,20]. In KOA, greater medial compartment loading and a higher external knee adduction moment (KAM) are associated with symptoms and disease severity [12,16], and hip muscle weakness—particularly of the abductors—is consistently observed, especially in medial-compartment KOA [27,43,44]. Hip abductor strengthening improves strength, pain, function, and 6 min walk performance [29,31], which substantiates the rationale for the present CKC and OKC programs focused on hip abduction and external rotation.

Biomechanically, CKC exercise is performed in a weight-bearing condition with the feet fixed to the ground, whereby ground reaction forces (GRFs) are transmitted from the distal to the proximal segments; this facilitates co-contraction of the quadriceps, hamstrings, and gluteus medius and promotes maintenance of joint alignment. These features help reduce excessive shear and suppress dynamic valgus collapse, thereby easing transfer to functional tasks such as level walking and stair negotiation [24,27,28,29]. Performance on the Y-Balance Test (YBT) is closely associated with hip strength—particularly abductor function—providing a mechanistic rationale for the superiority of hip-focused CKC in improving dynamic balance [33]. By contrast, OKC training in non-weight-bearing settings is effective for isolated strengthening of target muscles but shows limited transfer to activities that demand multidirectional loading and postural control [9,24].

In the affected-limb analysis of the present study, the CKC group exhibited significant reductions across all COP variables—range, distance, and velocity in both the anterior–posterior (AP) and medial–lateral (ML) directions, indicating improved postural stability. Because KOA commonly involves affected-side muscle weakness, joint instability, and proprioceptive deficits that directly degrade balance [8,17,18], the CKC mechanisms of co-contraction and alignment control appear to counter these functional limitations under weight-bearing conditions [27,28,29].

On the contralateral side, the CKC group likewise demonstrated significant decreases in COP variability, suggesting that CKC benefits extend beyond recovery of the affected limb to improved neuromuscular control of the non-affected limb, where overreliance and compensatory loading often emerge in unilateral KOA gait [43,45]. In this context, the bilateral participation inherent to CKC tasks may foster symmetry restoration and system-wide reorganization of balance control [27]. Moreover, a recent systematic review concluded that kinetic-chain strengthening enhances intersegmental force transmission and whole-body coordination, thereby promoting functional recovery [46]. Consistently, a randomized clinical trial in older women reported that multisensory plus CKC training produced meaningful gains in functional performance and balance [47].

Dynamic balance (YBT) improved significantly in the CKC group for both the affected and unaffected limbs, with a larger gain on the affected side. This pattern suggests that CKC concurrently stimulates lower-extremity strength and proprioception, thereby enhancing dynamic balance grounded in whole-body coordination, consistent with the reported association between YBT performance and hip strength [33]. Because dynamic postural control is linked to activities of daily living (ADL), quality of life, and reduced fall risk [18], the observed YBT improvements with CKC are clinically meaningful for functional independence and safety. Taken together, these findings imply that the mechanistic advantages of hip abductor/external rotator-focused CKC—namely, promotion of multi-joint coordination and co-contraction under weight-bearing conditions—can translate into superior clinical outcomes [24,25,27,28,29,33].

By contrast, the OKC group showed no significant YBT change, although small improvements were noted in several COP variables. This pattern indicates that OKC may contribute to modest reductions in sway during static tasks (i.e., single-leg quiet stance) by selectively strengthening target muscles such as the hip abductors, external rotators, and flexors [9,24]. Nevertheless, in the post-test between-group comparisons, the CKC group consistently exhibited lower AP, ML, Total distance, and velocity indices, underscoring the superiority of CKC for postural stability and transfer to functional balance under multiplanar, weight-bearing demands. Accordingly, for KOA rehabilitation where balance and gait stability are primary goals, hip abductor/external rotator-focused CKC should be prioritized over OKC as the clinical option of choice [24,25,27,28,29,33].

In summary, the present study provides objective, index-based evidence that hip abduction/external rotation-focused CKC comprehensively improves both static (COP) and dynamic (YBT) balance in middle-aged women with KOA, while promoting recovery of symmetry between the affected and unaffected limbs and enhancing gait-related stability. Although OKC retains a role for isolated strength gains and symptom management [9,24], when the therapeutic objective is functional recovery predicated on multi-joint, weight-bearing control, CKC represents the more effective strategy [12,16,27,28,29,33].

This study has several limitations. First, regarding mechanistic outcomes, we limited measurement to static and dynamic balance (COP and YBT), and did not capture joint kinetics (joint moments, GRF), muscle activation (EMG), or joint contact forces. Future trials should integrate simultaneous 3D motion–GRF–EMG acquisition and musculoskeletal modeling to elucidate neuromuscular and mechanical mechanisms.

Second, the sample size and assessment procedures impose constraints. With a relatively small cohort, we cannot exclude underestimation of group × time interactions; moreover, full blinding and complete standardization of testing conditions (e.g., foot position, gaze, footwear, breathing) were not achieved, which may have introduced condition sensitivity into the outcomes. Larger, a priori-powered, multi-center trials with assessor/data-analyst separation and tighter protocol standardization are warranted.

Third, external validity and durability were not fully addressed. Participants were middle-aged women with KOA, which limits the results’ generalizability, and the 10-week intervention was not followed by longitudinal assessments, so the persistence of effects remains unknown. Stratified analyses by sex, age, body composition, alignment, and disease severity (e.g., KL grade), along with 3–6-month follow-up and comparison of maintenance/booster protocols, are needed to verify long-term effectiveness and optimize clinical implementation.

## 5. Conclusions

In middle-aged women with knee osteoarthritis, a 10-week closed-kinetic-chain (CKC) program emphasizing hip abduction and external rotation produced broader and more consistent improvements in both static balance (COP) and dynamic balance (YBT) than an open-kinetic-chain (OKC) program. Under weight-bearing conditions, CKC promotes multi-joint coordination and co-contraction, supporting joint alignment; in our cohort, this translated to significant reductions in COP distance and velocity and meaningful gains in YBT performance changes that are clinically relevant to restoring postural stability for gait and functional tasks [24,25,27,28,29,33].

Although the OKC group exhibited small improvements in select COP variables, dynamic balance did not improve significantly, indicating that isolated strengthening in non–weight-bearing positions has limited transfer to multiplanar, postural control-demanding activities [9,24]. Collectively, these findings support prioritizing hip abduction/external rotation–focused CKC as a first-line exercise strategy when rehabilitation goals include everyday function and gait stability in KOA [24,25,27,28,29,33]. Future studies should refine CKC dosing, intensity, and progression while incorporating mechanistic metrics, larger and more diverse samples, and longitudinal follow-up to strengthen causal inference and inform clinical guidelines.

## Figures and Tables

**Table 1:** Characteristics of study participants (n = 22).

	OKC (n = 11)	CKC (n = 11)
Age (year)	62.1 ± 2.52	63.6 ± 1.80
Weight (kg)	59.5 ± 7.40	62.3 ± 6.45
Height (cm)	157.36 ± 6.36	160.86 ± 5.94
BMI (kg/m^2^)	24.04 ± 2.43	24.10 ± 2.62

Values are presented as mean ± standard deviation. OKC: control group; CKC: experimental group.

**Table 2:** CKC group protocol (10 weeks; 2 sessions per week).

CKC Group	Exercise
Warm-up (Mobility for 10 min)	- Ankle and hip mobility
Main exercise (for 40 min/3 set)	- The low diagonal oblique sit - Side plank AB drop up/down - Single-leg squat with isometric hip abduction (gym ball) - The hip airplane
Cool-down (stretching for 10 min)	- Ankle and hip stretching

**Table 3:** OKC group protocol (10 weeks, with 2 sessions per week).

OKC Group	Exercise
Warm-up (Mobility for 10 min)	- Ankle and hip mobility
Main exercise (for 40 min/4 set)	- Side-lying hip abduction - Side-lying hip external rotation - Seated hip external rotation - Standing hip abduction
Cool-down (stretching for 10 min)	- Ankle and hip stretching

## Data Availability

The data used to support the findings of this study are included in the manuscript.

## Abbreviations

The following abbreviations are used in this manuscript:

KOA	Knee osteoarthritis.
CKC	Closed-kinetic-chain exercise.
OKC	Open-kinetic-chain exercise.
COP	Center of pressure.
AP	Anterior–posterior.
ML	Medial–lateral.
RMS	Root mean square.
GRF	Ground reaction forces.
YBT	Y-balance test.
